# The Association of Hippocampal Long-Term Potentiation-Induced Gene Expression with Genetic Risk for Psychosis

**DOI:** 10.3390/ijms25020946

**Published:** 2024-01-12

**Authors:** Natalie L. Wellard, Nicholas E. Clifton, Elliott Rees, Kerrie L. Thomas, Jeremy Hall

**Affiliations:** 1 Division of Psychological Medicine and Clinical Neurosciences, Cardiff University, Cardiff CF24 4HQ, UKreeseg@cardiff.ac.uk (E.R.); thomaskl5@cardiff.ac.uk (K.L.T.); hallj10@cardiff.ac.uk (J.H.); 2 Medical School, Faculty of Health and Life Sciences, University of Exeter, Exeter EX4 4QF, UK

**Keywords:** long-term potentiation, gene expression, psychosis, schizophrenia, bipolar disorder, genomics, association analysis, learning

## Abstract

Genomic studies focusing on the contribution of common and rare genetic variants of schizophrenia and bipolar disorder support the view that substantial risk is conferred through molecular pathways involved in synaptic plasticity in the neurons of cortical and subcortical brain regions, including the hippocampus. Synaptic long-term potentiation (LTP) is central to associative learning and memory and depends on a pattern of gene expression in response to neuronal stimulation. Genes related to the induction of LTP have been associated with psychiatric genetic risk, but the specific cell types and timepoints responsible for the association are unknown. Using published genomic and transcriptomic datasets, we studied the relationship between temporally defined gene expression in hippocampal pyramidal neurons following LTP and enrichment for common genetic risk for schizophrenia and bipolar disorder, and for copy number variants (CNVs) and de novo coding variants associated with schizophrenia. We observed that upregulated genes in hippocampal pyramidal neurons at 60 and 120 min following LTP induction were enriched for common variant association with schizophrenia and bipolar disorder subtype I. At 60 min, LTP-induced genes were enriched in duplications from patients with schizophrenia, but this association was not specific to pyramidal neurons, perhaps reflecting the combined effects of CNVs in excitatory and inhibitory neuron subtypes. Gene expression following LTP was not related to enrichment for de novo coding variants from schizophrenia cases. Our findings refine our understanding of the role LTP-related gene sets play in conferring risk to conditions causing psychosis and provide a focus for future studies looking to dissect the molecular mechanisms associated with this risk.

## 1. Introduction

Genomic studies have provided critical insights into the molecular pathways contributing to risk for neuropsychiatric disorders. There is increasing evidence that many susceptibility genes for schizophrenia and related disorders impact synaptic plasticity mechanisms [1,2]; however, the nature of these associations, including the specific genes, physiological processes, cell types and timepoints implicated, remain to be further clarified.

In psychiatric genomics, genetic variants conferring risk are typically placed into one of two categories based on their prevalence: common variants with individually low effect sizes, typically identified through genome-wide association studies (GWASs), and rare variants, often with larger effect sizes, including copy number variants (CNVs) and rare coding variants. An extended analysis of the latest and largest schizophrenia GWAS meta-analysis from the Psychiatric Genomics Consortium (PGC3) identified common variants associated with schizophrenia in 287 distinct genomic loci [3]. Further genomic studies have identified rare CNVs at several loci that associate with schizophrenia, including deletions at 1q21.1, 3q29, 22q11.2 and *NRXN1*, and duplications at 16p11.2 [4,5,6,7,8]. Deleterious rare coding mutations associated with schizophrenia, including those arising de novo, have been identified through the exome sequencing of cases and controls [9,10,11].

Several of the genetic variants identified through common and rare variant analyses of schizophrenia impact synaptic processes [1]. In 2012, Kirov and colleagues combined CNV and proteomics datasets to conduct a systematic analysis of synaptic protein complexes [12]. They found an enrichment of post-synaptic density (PSD) genes within schizophrenia-associated CNVs, including a particular enrichment of genes involved in the N-methyl-D-aspartate (NMDA) receptor complex and activity-regulated cytoskeleton-associated protein (Arc) interactors. A further larger investigation confirmed the association between patient CNVs with NMDA receptor- and Arc-associated genes, and also found that CNVs were enriched more broadly for other regulators of synaptic function, including GABA receptors and calcium channels, and genes activated during synaptic plasticity and associative learning [13]. Recent evidence from a Schizophrenia Exome Meta-Analysis (SCHEMA) consortium study also implicates protein-damaging mutations affecting synaptic genes, including the NMDA receptor subunit *GRIN2A*, the AMPA receptor subunit *GRIA3* and a synaptic voltage-gated calcium channel (*CACNA1G*) [10]. Large-scale common variant (GWAS) studies have also strongly implicated genes encoding synaptic proteins in risk for schizophrenia [3] and bipolar disorder [14,15]. Importantly, in some cases, converging evidence from both common and rare variant studies implicates the same synaptic genes, including *GRIN2A* in schizophrenia [1,3,10].

Through the use of curated databases such as Gene Ontology (GO), previous genomic studies have suggested an enrichment of long-term potentiation (LTP)-induced genes for genetic risk associated with schizophrenia mediated through both common variants [16] and rare copy number variants [13]. First described by Bliss and Lomo at excitatory synapses in the hippocampus [17], LTP is a key form of activity-dependent plasticity, giving rise to persistent increases in synaptic strength and connectivity that underly associative learning and memory [18]. While there are multiple forms of LTP discernible by their stimulation requirements for induction and initial cellular mechanisms for expression, a key distinction is that persistent LTP (late LTP or LTP3) depends on protein synthesis, whereas early LTP (or LTP/LTP2) not sustained beyond a few hours does not [19,20]. There is a general consensus that long-term memory requires de novo mRNA and protein synthesis [21,22]. Patterns of de novo gene expression persist for hours after LTP induction [23], but at which timepoints or in which cell types schizophrenia risk variants impact LTP-associated molecular pathways are unknown.

Several approaches have been used to investigate hippocampal gene expression following the induction of LTP. Early studies focused on candidate gene expression; however, as technology progressed, transcriptome-wide studies began to identify more extensive lists of genes expressed following LTP, including those involved in transmitter transport, growth factors, ion channels, Arc interactors and regulation of the cytoskeleton [24,25,26,27,28]. In particular, the advent of Translating Ribosome Affinity Purification (TRAP) has allowed for the profiling of translating RNA from select populations of cells [29,30]. TRAP by RiboTag [31] involves genetically tagging a ribosomal protein under the control of a tissue-specific promotor and isolating ribosome-associated mRNAs by immunoprecipitation for transcriptome profiling. Chen and colleagues (2017) utilized this technique to profile the genes expressed 30, 60 and 120 min after chemically induced LTP in hippocampal slices [23] (Supplementary Figure S1). The ribosome tag was driven by a αCaMKII promotor [32] to profile LTP gene expression specifically in CA1 excitatory pyramidal neurons, a cell type genetically associated with schizophrenia through their unique repertoire of gene expression [33]. They highlight a range of synaptic and non-synaptic pathways stimulated by LTP and present specific sets of genes expressed at each timepoint.

Here, we explored the relationship between common and rare genomic risk factors for schizophrenia and related disorders with genes expressed at different timepoints after LTP induction in mouse hippocampal CA1 excitatory neurons [23].

## 2. Results

### 2.1. Common Variant Association

**Figure 1:**
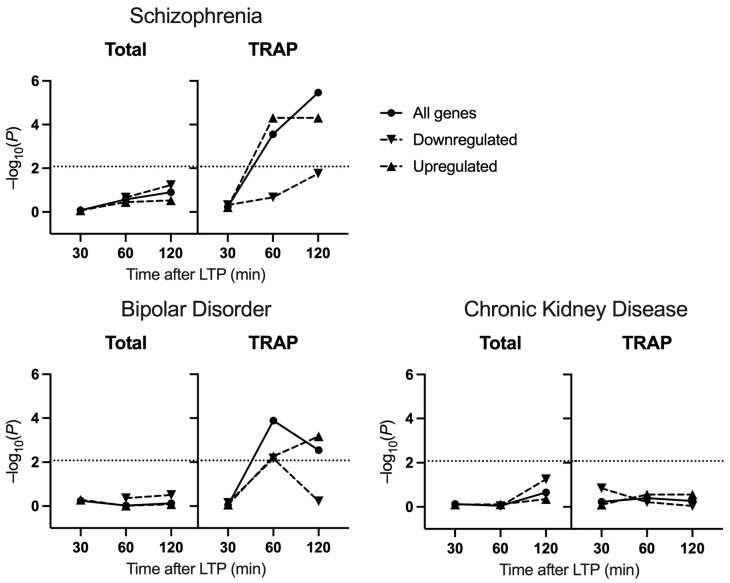
Gene set enrichment analysis of LTP datasets for common variant association with schizophrenia, bipolar disorder, and chronic kidney disease (negative control). Gene sets represent differentially expressed genes at 30, 60 and 120 min following LTP compared to baseline expression in the hippocampus. *p*-values have been log10 transformed. Dotted lines represent the threshold for statistical significance after correction for multiple testing.

Transcriptomic changes following LTP were obtained from published datasets derived from mouse hippocampal CA1 using total RNA-seq and cell-type-specific TRAP-seq [23]. To assess the enrichment in LTP-related genes for common genetic association with schizophrenia and related disorders, we used GWAS summary statistics to perform gene set enrichment analyses [34] on sets of genes captured at distinct timepoints following LTP (Supplementary Figure S1), conditioning on all tissue-expressed genes. There was a significant enrichment for association in genes identified through TRAP-seq at 60 and 120 min after LTP induction but not at 30 min (30 min: β = −0.032, Bonferroni-adjusted p (p.bonf) = 1.0; 60 min: β = 0.12, p.bonf = 0.0017; 120 min: β = 0.17, p.bonf = 2.1 × 10^−5^) (Figure 1). At both 60 min and 120 min, the association was driven by the upregulated subset (60 min: β = 0.18, p.bonf = 3.0 × 10^−4^; 120 min: β = 0.17, 2.9 × 10^−4^), whilst downregulated genes were not significantly enriched for association (60 min: β = 0.037, p.bonf = 1.0; 120 min: β = 0.17, p.bonf = 0.10). Among the differentially expressed genes (DEGs) derived from total RNA-seq, there was no evidence of enrichment for common schizophrenia risk at any time point (30 min: β = −0.23, p.bonf = 1.0; 60 min: β = 0.076, p.bonf = 1.0, 120 min: β = 0.067, p.bonf = 0.75).

Similarly, gene sets identified through TRAP-seq at 60 min and 120 min after LTP were also associated with bipolar disorder risk (TRAP-seq: 30 min: β = −0.17, p.bonf = 1.0, 60 min: β = 0.11, p.bonf = 7.8 × 10^−4^, 120 min: β = 0.095 p.bonf = 0.017). Whilst the association at 120 min was driven by upregulated genes (upregulated: β = 0.12, p.bonf = 0.0041; downregulated: β = −0.019, p.bonf = 1.0), the association at 60 min was prevalent in both up- and downregulated genes (upregulated: β = 0.10, p.bonf = 0.033; downregulated: β = 0.10, p.bonf = 0.041). When the two bipolar subtypes were analysed separately, we observed that the association signal from LTP-induced genes was restricted to bipolar subtype I (Table 1). In addition, there was no significant enrichment of any gene sets for common variant association with chronic kidney disease (TRAP-seq 30 min: β = −0.024, p.bonf = 1.0, 60 min: β = 0.0061, p.bonf = 1.0, 120 min: β = −0.0029, p.bonf = 1.0; Total RNA-seq 30 min: β = −0.11, p.bonf = 1.0, 60 min: β = −0.11, p.bonf = 1.0, 120 min: β = 0.033, p.bonf = 1.0).

### 2.2. Schizophrenia CNV Enrichment

**Figure 2:**
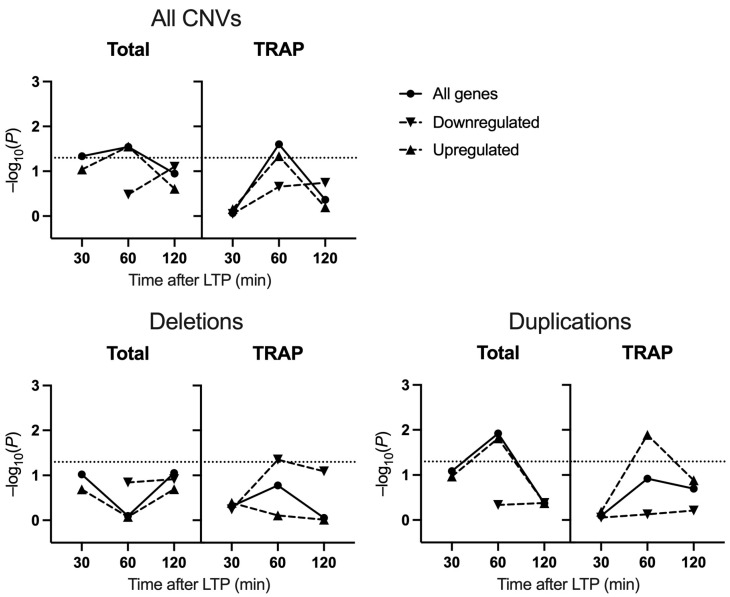
Enrichment of LTP gene sets in schizophrenia-associated CNVs. Logistic regression analysis was used to test the relative enrichment of each gene set in case CNVs compared to control CNVs. Deletions and duplicates were analysed together (All CNVs) and separately. *p*-values resulting from logistic regression analysis have undergone permutation correction to derive empirical *p*-values and adjust for inflation and background enrichment. Dotted lines represent the threshold for significance following permutation correction.

To investigate the enrichment of LTP gene sets in schizophrenia-associated CNVs, we performed CNV enrichment analysis using deletions and duplications from cases and controls. Similar to the common variant enrichment, genes expressed 60 min post-LTP in CA1 excitatory neurons captured by TRAP were found to be enriched in CNVs from patients (β = 0.12, p.unadjusted = 8.75 × 10^−6^, p.adjusted = 0.025) (Figure 2), again driven by the upregulated subset (β = 0.14, p.unadjusted = 1.4 × 10^−4^, p.adjusted = 0.046). Of genes deriving from total RNAseq, those differentially expressed at 30 min and 60 min were also enriched in case CNVs (30 min: β = 1.12, p.unadjusted = 0.0063, p.adjusted = 0.046; 60 min: β = 0.55, p.unadjusted = 5.1 × 10^−4^, p.adjusted = 0.029) (Figure 2).

Deletion and duplication CNVs were then analysed separately to investigate whether our results were driven by a particular subset of CNVs. Interestingly, only the 60 min downregulated gene set from TRAP-seq was significantly enriched for deletions (β = 0.25, p.unadjusted = 2.0 × 10^−5^, p.adjusted = 0.045), whilst the 60 min upregulated genes from TRAP-seq or total RNA-seq were enriched for duplications (TRAP-seq: β = 0.21, p.unadjusted = 1.3 × 10^−6^, p.adjusted = 0.013; total RNA-seq: β = 0.96, p.unadjusted = 6.8 × 10^−7^, p.adjusted = 0.016).

### 2.3. Schizophrenia De Novo Rare Coding Variant Enrichment

Lastly, the enrichment of de novo rare coding variants from schizophrenia cases in the LTP gene sets was examined, focusing on loss-of-function protein truncating variants (PTVs) and missense mutations with an MPC score of greater than 2. TRAP-acquired genes induced 120 min after LTP were enriched for case variants of these classes with nominal significance, but there was no significant enrichment of case variants in the TRAP- or total RNA-seq conditions when corrected for multiple comparisons (Table 2).

## 3. Discussion

In the present study, we tested sets of LTP-related genes for genetic association with schizophrenia and bipolar disorder. The analysis of transcriptomes from total tissue and CA1 pyramidal neurons specifically, and at multiple experimental timepoints, allowed for an in-depth investigation into the manner in which psychiatric risk variants impact on LTP-induced genes. Our primary finding was an enrichment of LTP-induced genes from 60 and 120 min timepoints in common genetic variants for both schizophrenia and bipolar disorder type I and also an enrichment of LTP-induced genes for CNVs in schizophrenia.

Previous studies of psychiatric genomics found an association between schizophrenia risk and genetic pathways implicating synaptic function, including LTP-related gene sets [3,5,12,13,16,35]. Here, we expand on these findings by demonstrating the temporal, spatial and directional specificity of these associations. We report that there was an enrichment of LTP-associated genes for association with schizophrenia, as conferred through common variation, at 60 and 120 min post-LTP-induction. This association was restricted to upregulated genes and was only identified using data from tissue-specific TRAP-seq and not that from total hippocampal samples, suggesting that LTP-regulated transcripts implicated in schizophrenia through common variant association are specifically enriched in CA1 pyramidal neurons. These results are consistent with previous studies, showing that CA1 pyramidal cells are impacted by schizophrenia risk variants [33]. Furthermore, the enrichment of schizophrenia risk variants, particularly those with synaptic annotations, in mouse and human CA1 [3] and in LTP-regulated transcripts in CA1 (here) highlight the functional impact of the risk variants on plasticity mechanisms underlying specific learning and memory functions in the mature brain. The selective enrichment highlights the benefit of using the tissue-specific TRAP method to interrogate LTP-related transcriptomics and identify a localised association in a functional setting.

The transition to persistent forms of LTP and from short-term to long-term memory involves the transcription of genes and proteins that are associated with synaptic growth [36,37]. The distinctive, temporal progression of gene expression measured by either total RNA seq or TRAP-Seq by Chen and colleagues (2017) brings to light the co-ordinated programme of gene expression triggered by LTP induction. These data are consistent with the early regulation of genes such as transcription factors that have effector roles in the subsequent expression of genes, supporting the synaptic structural changes associated with long-term synaptic plasticity and memory storage [36]. The enrichment of LTP-associated genes for association with schizophrenia at 60 and 120 min but not the earliest time point investigated concurs with the observation that synaptic proteins, consequential for maintaining plasticity and memory, are also enriched for association with schizophrenia [3]. It is possible that the impact of schizophrenia risk variants on adult plasticity may preferentially affect the magnitude and persistence of synaptic changes after induction or following learning.

Gene expression captured in CA1 pyramidal neurons 60 and 120 min following LTP induction was similarly associated with risk for bipolar disorder. Furthermore, when the GWAS data were analysed separately for each bipolar disorder subtype, we observed that the association was restricted to bipolar I cases. Clinically and genomically, bipolar disorder type I is more strongly related to schizophrenia, consistent with their shared association with LTP-induced genes, whereas bipolar disorder II is more aligned with the clinical and genomic characteristics of major depressive disorder [14,15,38]. Common variants associated with bipolar disorder were recently found to be enriched in genes expressed in the hippocampus, including hippocampal pyramidal neurons, and in GO terms relating to synaptic signalling [15]. Thus, the current finding adds to the growing literature implicating dynamic synaptic processes in the aetiology of both schizophrenia and bipolar disorder, although we note that genetic discovery sample sizes were larger for bipolar disorder type I than bipolar disorder type II, potentially limiting power to identify associations with bipolar disorder type II. By testing for associations in a chronic kidney disease GWAS of comparable sample size to the schizophrenia and bipolar disorder GWAS, we provide further confidence that our primary results were not inflated by methodological artefacts of using large GWAS samples.

To further investigate the relationship between LTP and schizophrenia genetic risk, we examined the enrichment of LTP-induced genes in case CNVs. Previously, CNVs in people with schizophrenia were shown to be enriched for genes involved in synaptic plasticity, including those encoding Arc interactors and NMDA receptor complexes, and genes associated with abnormal synaptic transmission and LTP [12,13]. In combined analyses of both deletion and duplication CNVs, an enrichment of genes upregulated 60 min after LTP was observed. The enrichment was mirrored in gene sets derived from both total and TRAP RNA-seq. This could reflect a lack of specificity of the risk conferred by CNVs to CA1 pyramidal neurons, instead impacting a range of hippocampal cell types involved in LTP induction. Alternatively, the enrichment in case CNVs may be driven by a small number of genes with large expression changes in pyramidal neurons, detectable in total RNA-seq. By separating analyses of CNV subtypes, we found that the enrichment of LTP-induced genes in case CNVs was particularly driven by duplications.

Taken together, these results suggest that genetic risk for schizophrenia, as conferred through CNVs, impacts a different subset of LTP-related genes compared to common variants. The difference is firstly highlighted by the temporal pattern of enrichment, whereby CNVs (duplications) were enriched for genes expressed at 60 min but not 120 min. Whilst 60 min was also characterised by the largest gene set, permutation analyses adjusted for this. Secondly, the specificity of the genetic association to TRAP-derived gene sets observed in analyses of GWAS data was not seen in tests of CNV enrichment. Considering previous findings demonstrating a contribution of genes involved in inhibitory synaptic complexes to the risk associated with CNVs [13], our results may reflect differences in the contribution of inhibitory neurons to the association signals derived from case CNVs vs. common variants. However, some of the association attributable to CNVs may be missed through gaps in probe coverage and filtering of CNVs by size, which could remove the signal from some genes also impacted by common variants.

Further inconsistencies across types of genetic variant emerged when examining the association of LTP-related genes sets with schizophrenia through de novo coding variants. We found only nominal enrichment for de novo coding variants from schizophrenia probands in LTP-induced genes at 120 min from TRAP but no overall association regardless of timepoint or transcriptomic approach. Previous research reported that de novo nonsynonymous mutations from patients with schizophrenia are enriched in synaptic gene sets, the postsynaptic density and associated protein complexes [39]. That we observe no such enrichment in LTP sets suggests that these variants confer risk through the disruption of synaptic pathways other than those related to potentiation. However, the current study of de novo variation might be underpowered to detect these effects. Increased sample sizes from exome sequencing will contribute to increased confidence in understanding how de novo coding variants play a role in modulating plasticity mechanisms in schizophrenia.

Our interpretations of these data assume that post-LTP transcriptomic changes in humans sufficiently mirror those observed in mice. Whilst there may be small differences in the temporal patterns or precise transcripts expressed following LTP, the composition and function of neuronal synapses are strongly conserved between the species [40,41]. Since our conclusions are based on genetic associations of groups of co-expressed genes and not individual genes, minor effects of species are not expected to alter our main findings.

Our findings build on our understanding of genetic associations in LTP-related gene sets in schizophrenia and bipolar disorder by refining these associations according to their temporal expression patterns, cell types and classes of genomic risk variant. In the case of bipolar disorder, we provide additional specificity to the disorder subtype in which the associations manifest, supporting an association specifically with bipolar disorder type I. The data highlight a reduced set of LTP-induced genes for the focus of downstream studies into plasticity mechanisms relevant to the development of psychiatric disorders.

## 4. Materials and Methods

### 4.1. LTP-Induced Gene Sets

RNA-sequencing datasets were generated by Chen and colleagues [23]. Briefly, RiboTag mice were generated with a floxed HA-tagged ribosomal protein L22 (The Jackson Laboratory: 011029) under CaMKIIα-cre mediated expression (The Jackson Laboratory: 005359) to direct expression to the CA1 pyramidal cell layer of the hippocampus. At 8–12 weeks old, a chemical LTP induction protocol was applied to ex vivo hippocampal slices with the dentate gyrus (DG) removed, using forskolin [42,43]. Chen and colleagues sequenced ribosome-bound mRNA from CA1 excitatory neurons and total mRNA from bulk hippocampal tissue at baseline and at three timepoints after LTP induction (30 min, 60 min and 120 min).

Processed RNA sequencing datasets were obtained from the NCBI Gene Expression Omnibus (GEO), accession number GSE79790. These datasets contained the log fold change (logFC) and false-discovery rate (FDR) for each gene, for each contrast of interest. These contrasts were: “TRAP-LTP vs. TRAP-basal” (TRAP-seq) and “Total-LTP vs. Total-basal” (total RNA-seq), for each of the three time points. These datasets were further filtered to include only those genes with an FDR < 0.01 to form the gene sets. Note that this significance threshold is more stringent than that used in the original study [23]. To facilitate comparisons with human data, mouse Entrez IDs were converted to their human homologs. Genes that did not have a unique human homolog were excluded from further analyses.

After filtering of total RNA-seq data, there were 21 differentially expressed genes (DEGs) at 30 min, 97 DEGs at 60 min and 474 DEGs at 120 min following LTP. From TRAP-seq data, there were 67 DEGs at 30 min, 1499 DEGs at 60 min and 1082 DEGs at 120 min (Supplementary Figure S1).

### 4.2. Genotype Data

SNP genotype data were obtained from published case and control samples. Schizophrenia summary statistics were acquired from the primary meta-analysis from wave 3 of the psychiatric genomics consortium, using genotype data from 74,776 case and 101,023 control subjects of European, East Asian, African American and Latino ancestry [3,44]. Summary statistics for bipolar disorder, and each subtype, were acquired from a recent study of 41,917 cases and 371,549 controls of European ancestry [15]. A bipolar disorder type I meta-analysis was performed using 25,060 cases and 449,978 controls, and a bipolar disorder type II meta-analysis using 6781 cases and 364,075 controls [15]. Lastly, GWAS summary statistics from a study of chronic kidney disease, used for control purposes, derived from a meta-analysis of 41,395 cases and 439,303 controls of European ancestry [45].

Ultra-rare de novo coding variants from 3444 schizophrenia proband parent trios were obtained from published exome sequencing studies [46,47,48,49,50,51,52,53,54]. Protein truncating variants (PTVs), defined as frameshift variants, stop-gain variants and donor/acceptor splice site variants, as well as missense variants with a “Missense badness, Polyphen-2, constraint” (MPC) score of 2 or greater, were taken forward for analysis [55].

Copy number variants, including deletions and duplications, from schizophrenia cases and controls were collated from CLOZUK and Cardiff cognition in Schizophrenia samples [6,8], International Schizophrenia Consortium [56] and Molecular Genetics of Schizophrenia [57] samples, totalling 17,565 cases and 24,830 control subjects.

### 4.3. Gene Set Enrichment Analysis

Gene set enrichment analysis for common variation was undertaken using Multi-marker Analysis of GenoMic Annotation (MAGMA) [34]. GWAS summary statistic SNP files were filtered to remove SNP with an info score of less than 0.8 or an allele frequency of less than 1%. SNPs were mapped to genes using the NCBI build 37 gene location files as a reference, and gene-wide analysis performed with linkage disequilibrium calculated using the 1000 genomes reference file (European ancestry). The resulting gene-wide *p* values were used in gene set enrichment analyses, with all expressed genes included as a covariate within the model to control for any background association from brain-expressed genes. Expressed genes were defined independently for TRAP and total RNA-seq datasets and were defined as having a normalised expression count greater than 10 in at least 3 basal samples. Gene set *p*-values were subjected to Bonferroni correction where appropriate.

De novo coding variant gene set enrichment was determined using a two sample Poisson rate ratio test, which compared the observed rate of variants in the gene set with the expected rate under the null (https://github.com/reeseg/SZ_NDD_pleiotropy_analysis (accessed on 8 March 2023)), while correcting for the background enrichment of these variants from brain-expressed genes. The expected number of de novo coding variants was estimated from per-gene mutation rates [58].

CNV datasets were filtered to only include those larger than 100 kb in size and covered by at least 15 probes. Protein coding genes overlapping each CNV were identified using the NCBI build appropriate to the dataset (ISC: build 35, MGS: build 36, CLOZUK: build 37), and the number of genes overlapping each CNV was counted. Logistic regression analysis was used to investigate the relative enrichment of a set of genes in case CNVs compared to controls using a general linear model:

Case–control status _~_ number of genes in CNV + CNV size + number of probes + CNV study + number of gene set hits

To adjust for *p*-value inflation with larger gene set sizes, permutation correction was applied. The background gene set was permutated 2000 times to generate a null distribution of *p*-values from random size-matched sets of brain-expressed genes. This procedure yielded an empirical *p*-value for each LTP gene set.

## Figures and Tables

**Table 1:** Enrichment for common variant association with bipolar disorder, split by bipolar disorder subtypes (I and II). Results obtained from gene set enrichment analyses in MAGMA, conditional on all tissue-expressed genes. Bold text indicates significant enrichment of gene set. *p*-values were Bonferroni adjusted to account for testing of multiple gene sets. SE = Standard error.

	Bipolar Disorder Subtype	β Value (SE)	*p*-Value	Adjusted *p*-Value
30 min TRAP-seq	Bipolar I	−0.090 (0.13)	0.76	1.0
	Bipolar II	−0.090 (0.11)	0.80	1.0
30 min total RNA-seq	Bipolar I	−0.066 (0.22)	0.62	1.0
	Bipolar II	−0.11 (0.18)	0.72	1.0
60 min TRAP-seq	**Bipolar I**	**0.13 (0.029)**	**3.1 × 10^−6^**	**1.9 × 10^−5^**
	Bipolar II	0.0025 (0.025)	0.46	1.0
60 min total RNA-seq	Bipolar I	−0.027 (0.10)	0.60	0.898
	Bipolar II	−0.096 (0.091)	0.85	1.0
120 min TRAP-seq	**Bipolar I**	**0.094 (0.034)**	**0.0027**	**0.016**
	Bipolar II	0.0070 (0.029)	0.40	1.0
120 min total RNA-seq	Bipolar I	−0.028 (0.050)	0.71	1.0
	Bipolar II	−0.028 (0.043)	0.74	1.0

**Table 2:** LTP gene sets were tested for enrichment of de novo protein truncating variants (PTVs) or missense variants with an MPC score of greater than 2 from schizophrenia cases. Adjusted *p*-values have been corrected for background enrichment in tissue-expressed genes and multiple comparisons using the Bonferroni method.

LTP Gene Set	Schizophrenia Ultra-Rare Coding Variant Enrichment			
	Observed/Expected	*p*-Value	Adjusted *p*-Value	Rate Ratio (95% Confidence Intervals)
30-min total	2/0.497	0.116	0.736	3.45 (0.416–12.5)
30-min TRAP	5/3.45	0.610	1.00	1.24 (0.401–2.91)
60-min total	7/3.27	0.114	0.642	1.84 (0.737–3.82)
60-min TRAP	80/57.4	0.101	0.931	1.23 (0.954–1.56)
120-min total	17/14.2	0.900	1.00	1.02 (0.592–1.66)
120-min TRAP	80/51.4	0.00892	0.181	1.39 (1.08–1.77)

## Data Availability

Data are contained within the article and cited works.

## Supplementary Materials

The supporting information can be downloaded at: https://www.mdpi.com/article/10.3390/ijms25020946/s1.
