# Supplementary material for: The Association of Hippocampal Long-Term Potentiation-Induced Gene Expression with Genetic Risk for Psychosis
Source: Int J Mol Sci. 2024 Jan 12;25(2):946. doi: 10.3390/ijms25020946 (PMC10816085; doi:10.3390/ijms25020946)
Supplement: Supplementary file 1 [file ijms-25-00946-s001.zip › ijms-2783955-supplementary.pdf]

The Association of Hippocampal Long-Term Potentiation-Induced Gene Expression with Genetic Risk for Psychosis

Natalie L. Wellard [1], Nicholas E. Clifton [1,2,*], Elliott Rees [1], Kerrie L. Thomas [1] and Jeremy Hall [1]

[1] Division of Psychological Medicine and Clinical Neurosciences, Cardiff University, Cardiff CF24 4HQ, UK; reeseg@cardiff.ac.uk (E.R.); thomaskl5@cardiff.ac.uk (K.L.T.); hallj10@cardiff.ac.uk (J.H.)
[2] Medical School, Faculty of Health and Life Sciences, University of Exeter, Exeter EX4 4QF, UK
* Correspondence: n.clifton@exeter.ac.uk

Supplementary Information

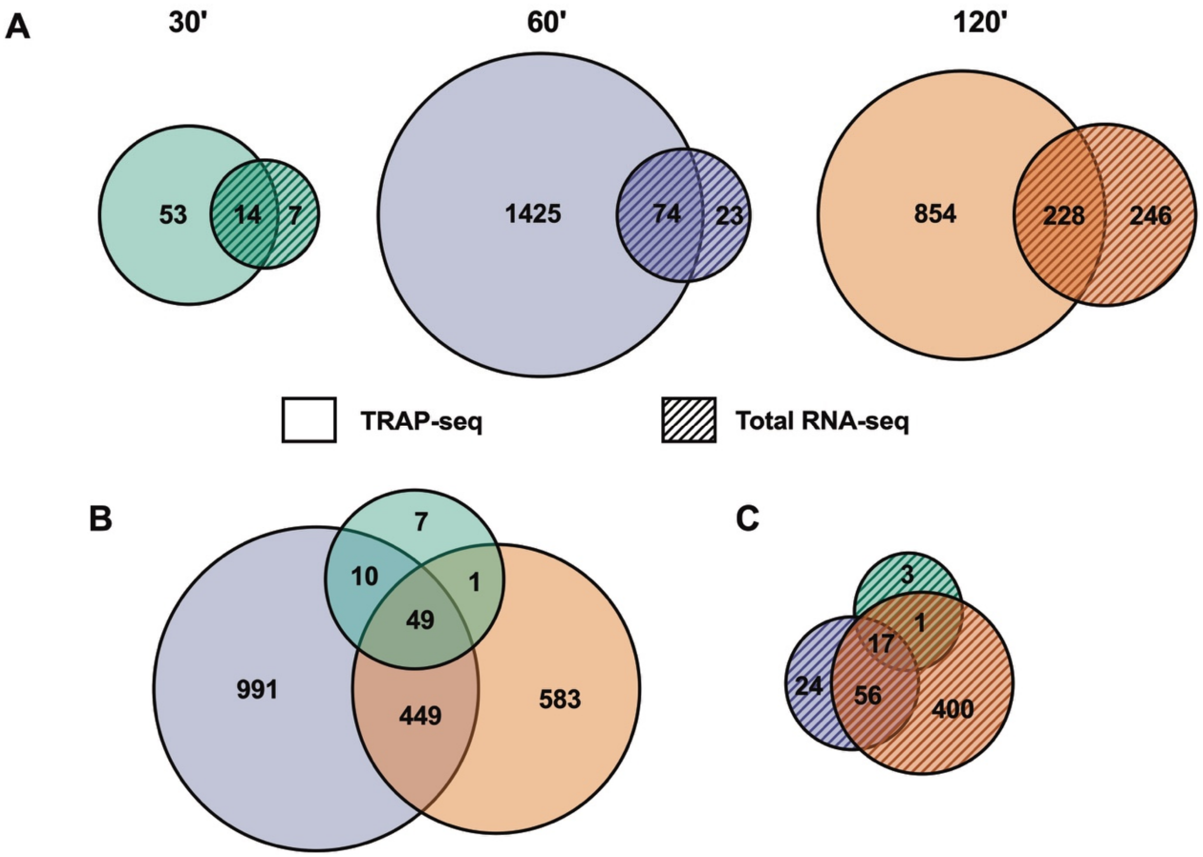

Supplementary Figure S1 The intersection between genes differentially expressed (FDR < 0.01) in the TRAP and total sequencing gene sets. A. The number of genes differentially expressed at 30 min (green), 60 min (purple) and 120 min (orange). B. The overlap between genes differentially expressed in the TRAP-seq condition across time points. C. The overlap between genes differentially expressed in the total RNA-seq condition across time points. Solid fill represents the TRAP-seq condition, patterned fill represents the total RNA-seq condition. Not to scale.
